# Supplementary material for: Study on deformation of tunnel pile foundation based on discrete element method and finite difference method
Source: PLoS One. 2024 Jul 18;19(7):e0307405. doi: 10.1371/journal.pone.0307405 (PMC11257389; doi:10.1371/journal.pone.0307405)
Supplement: Attachment — Submitted filename: Response to Reviewers.docx [file pone.0307405.s002.docx]

**Dear Editors and Reviewers:**

We sincerely appreciate the valuable feedback from the editor and all the reviewers, which has helped us improve the quality of the manuscript. Reviewer comments are listed below in italics and numbered for reference, and our responses are given in normal font. Changes/additions to the manuscript are indicated in red text.

**The questions are answered as follows:**

**Editor Comments:**

***1) A separated file must be provided for the authors&rsquo; answers to the comments, one by one. Moreover, all changes must be yellow-colored highlighted sentences in the revised article. The track changes condition is not suggested.***

**Response：**

All comments have been revised one by one according to the editor's opinion. Highlight the revised manuscript in yellow.

***2) No abbreviations must be used in the title.***

**Response：**

Modify the title to“Study on deformation of tunnel pile foundation based on discrete element method and finite difference method coupling”

***3) All abbreviations should be defined at first mentioning, such as DEM-FDM, etc.***

**Response：**

All abbreviations are defined at the first time when the article appears, for example, DEM-FDM has been defined in the abstract line 10 and the body line 68.

***4) No quantitative data could be seen in the abstract.***

**Response：**

Some expressions in the abstract were modified and the quantitative data were supplemented. The supplement has been highlighted in yellow.

***5) All keywords must be found in the abstract or the title.***

**Response：**

It has been verified that all keywords can be found in the summary or title.

***6) The introduction is lengthy. Moreover, the novelty must be highlighted in the introduction, compared to the literature review***

**Response：**

At the same time, the method innovation of this paper has been explained in the introduction, and the number of references has been supplemented. The changes have been highlighted in yellow.

***7) All used material properties need references, especially for the data in Tables 1 and 2.***

**Response：**

The data sources in Table 1 and Table 2 are laboratory experiments. As for the experimental sources of materials, this part has been supplemented in Chapter 2 of the paper.

***8) The structure is confusing. The text must have an introduction, research method, results and discussion, conclusions, and references.***

**Response：**

The article structure is adjusted from introduction, research method, results and discussion, conclusions, and references. Five parts have been described.

***9) Numerical results must be validated such as Figures 6 and 8.***

**Response：**

Since the displacement of pile foundation is difficult to collect in actual monitoring, this paper compares the actual monitoring of other collected data with the numerical simulation, so as to illustrate the reliability of the numerical simulation in this paper.

In this paper, the comparison of surface settlement curves has been used to verify the numerical simulation. The detailed verification content can be found from line234 to 246 of the revised draft.

***10) Conclusion must be changed to Conclusions. It is also lengthy. It must be shortened.***

**Response：**

According to the editor's opinion, the conclusions of the article have been simplified.

***11) The discussion is poor and it must be improved. They must be compared to other results of other similar articles.***

**Response：**

According to the editor's opinion, the discussion of the article has been simplified. In addition to this, comparisons with other articles have been added.

***12) References should be updated based on recent articles, published in 2015-2024. Moreover, it should be extended to at least 35 articles for a proper discussion.***

**Response：**

The references of the article have been supplemented to 36 articles.

**Reviewer 1:**

***This research paper addresses an important issue in engineering, specifically the deformation of pile foundations due to shield tunnel construction. The authors propose using the DEM-FDM coupling numerical simulation method to study the deformation response mechanism. This method appears to combine the discrete element method (DEM) and the finite difference method (FDM) to simulate the deformation process of pile foundations during tunnel driving. The paper focuses on three factors: the length of the pile foundation, the distance between the tunnel and the pile foundation, and the small distance between the three holes. The results indicate that the displacement and deformation response of adjacent pile foundations due to the construction of a three-hole tunnel with small spacing primarily involve horizontal displacement, bending, and inclination of the pile foundation.***

***Overall, this paper presents a valuable contribution to the field of geotechnical engineering but could benefit from further elaboration and contextualization.***

1. ***Did the researchers conduct any experimental validation or comparison with field data to verify the accuracy of their simulation results? It is suggested that researchers supplement or explain the comparative verification content.?***

**Response：**

Thank you very much for the valuable suggestions of the reviewers. The data sources of the numerical simulation in this paper mainly include the following three parts:

1. Laboratory tests: In the DEM field, the contact parameters of particles are calibrated through a series of large-scale triaxial test analyses. In the process of numerical simulation in this paper, the selection of parameters is subject to field monitoring, and the calibration test is carried out by triaxial test, and the modeling parameters of the following soils are obtained. Triaxial test is a common method to determine the macroscopic parameters of soil. Unconsolidated and undrained test is carried out on a series of sand and clay with density gradient through the triaxial tester. The relationship between the internal friction Angle ϕ, cohesion force c, deformation modulus k and porosity is fitted, and the correlation between the shear strength and porosity of the soil is studied, which provides a basis for the parameter assignment of the subsequent shield model.

**Triaxial analysis test**

2. Field observation and monitoring: This paper uses field observation and monitoring data to verify the accuracy of numerical simulation. This paper collects the actual data of the tunnel construction site, such as the change of ground settlement, pile foundation deformation, etc., and compares these data with the simulation results. The following is a comparison between the results of numerical simulation of land surface settlement change and the results of field monitoring. As can be seen from the figure below, the numerical model in this paper has a certain reliability, and the verification and comparison of the surface settlement values indicate that the model has a strong fitting ability.

3. Comparison of case studies: By comparing data from previous case studies of similar projects, researchers can assess the reliability of numerical simulation results. The above three comparison methods can verify the reliability of the numerical simulation.

2. ***Given the formation disturbance and horizontal soil deformation caused by the construction of the three-hole tunnel, are there any mitigation measures or design considerations proposed to minimize the negative effects on pile foundations?***

**Response：**

According to the opinions of the experts, in the conclusion part of the paper, the related mitigation measures caused by the three-hole tunnel are added to reduce the impact on the pile foundation, and the mitigation suggestions are put forward. At the same time, the discussion part of Section 4.4 is added to the revised manuscript to explain the mitigation of disturbance effects of tunnel construction on adjacent pile foundations. The specific content is elaborated in lines 494 to 522 of the revised manuscript, which is as follows:

**1. Geological investigation and analysis: carry out detailed geological investigation before construction to understand the formation conditions and geological characteristics. This helps to predict possible geological changes and the impact of tunnel construction on the soil layer.**

**2. Structural design optimization: By optimizing the structural design of the tunnel, the impact on the surrounding soil and pile foundation can be reduced. This may include adjusting the depth, width and shape of the tunnel to reduce formation disturbance.**

**3. Use appropriate construction techniques: Select appropriate construction techniques and methods, such as shield tunneling or other methods to reduce vibration and disturbance, to minimize the impact on the soil and pile foundation.**

**4. Formation reinforcement and support measures: Take formation reinforcement and support measures around the tunnel, such as the use of supporting piles, soil reinforcement or other geological engineering techniques to protect the pile foundation from disturbance.**

**5. Monitoring and adjustment: real-time monitoring during the construction process, and adjustment and control according to the monitoring results. This helps to identify problems in a timely manner and take necessary corrective actions to reduce negative impacts.**

**The above methods are helpful to reduce the negative effects of three-hole tunnel construction on pile foundation. In the actual project, it is necessary to consider the geological conditions, construction technology and design scheme to develop the best mitigation measures to reduce the adverse impact on the pile foundation and the surrounding soil.**

3. ***The results describe the displacement and deformation response of pile foundation under the influence of three-hole and small-distance tunnel construction, and the deformation response under the influence of three factors. However, when describing the results, it is recommended that more quantitative analysis be provided to support the accuracy and credibility of the conclusions.***

**Response：**

According to the expert opinion, the conclusion part of the article has been modified and simplified. The quantitative analysis under different factors is supplemented, and the revised conclusions are as follows:

**In this paper, the DEM-FDM coupling method was used to analyze the deformation response of pile foundation caused by three-hole tunnel excavation. The influence of the length of the pile foundation, the distance between the tunnel and pile foundation, and construction factors on displacement and stress were discussed. The conclusions are as follows:**

**(1) Influence on pile foundations of different lengths: It was found that when the distance between the pile foundation and tunnel was 3D, tunnel construction did not cause vertical displacement of the pile foundation. The difference in extreme vertical deformation caused by tunnel construction was not significant, and settlement displacement was concentrated in the range of 4.7cm to 4.9cm. The peak value of uplift was concentrated between 2.2cm and 2.5cm. The actual monitoring results and numerical simulation results were mutually verified.**

**(2) Influence on the distance between the tunnel and pile foundation: It was found that as the distance between the pile foundation and tunnel decrease, the pile mainly tilted. When the distance between the tunnel and pile foundation was 1D, the horizontal displacement difference between pile top and pile bottom was 8mm. It was found that the three lengths of the pile foundation showed a good linear correlation within 3D. However, theoretically, with the further increase in the distance between the tunnel and pile, the influence of tunnel construction on the pile foundation should gradually weaken and disappear.**

**(3) The influence of three-hole tunnel construction on pile foundation: It was found that the horizontal displacement of the pile top increased gradually with the penetration of three tunnels one by one. For H+2D pile foundation, the incremental horizontal displacement of the pile top caused by double-hole penetration was less than 0.5mm. However, when the three-hole tunnel was connected, the horizontal displacement increment of the pile top was relatively larger, about 1.5mm.**

4. ***This paper clearly points out the purpose of the study, that is, to investigate the deformation response mechanism of tunnel pile foundation caused by shield construction, and describes the numerical simulation method of DEM-FDM coupling. It is recommended to provide a more detailed introduction in the method section, including the steps of numerical simulation, parameter setting and model validation, so as to ensure that readers can fully understand the reliability and applicability of the research method.***

**Response：**

Detailed steps for additional numerical simulation and parameter setting, based on expert advice, can be found in line 164 -170 of the revised manuscript.

5. ***The readability of the manuscript is generally ok, but an editorial review by a native speaker must be conducted in places throughout the document.***

**Response：**

Thank you very much for your advice. We have carefully checked the entire article to avoid spelling, grammar and typographical errors in order to improve the readability and language quality of the article. Grammar or presentation issues that have been revised in the text are corrected in red in the revised manuscript. Thank you for your guidance and support!

**Reviewer 2:**

***This paper investigates the deformation response mechanism of pile foundation induced by the construction of three-hole small clear distance tunnel, by use of the DEM-FDM coupled numerical simulation method. Three factors, i.e., the length of pile foundation, the distance between tunnel and pile foundation, and the small clear distance of three-hole tunnel construction, have been focused on in this study. Some valuable results have been yielded; yet, the following issues should be clarified before it can be accepted for publication.***

1. ***The deformation study of adjacent pile foundation is mainly aimed at the deformation response induced by the construction of single tunnel or double tunnel. This study focuses the action mechanism and deformation response of the three-hole small distance tunnel construction to the pile foundation of adjacent buildings. Can authors provide some engineering background for the three-hole small distance tunnel construction near pile foundation?***

**Response：**

According to the expert opinion, this paper adds the engineering background of three-hole small-distance tunnel construction near pile foundation. This paper is based on a shield section of Nantong rail Transit Line 1. The section of the shield tunnel runs along the existing road. Along the line are the city's main roads and important commercial blocks and residential areas. The underground pipeline along the line is dense and passes through the existing civil air defense tunnel. The buildings and roads traversed by the shield section are shown in the figure below, which are supplemented in the line211-218 of the article.

(a) Project case site layout

(b) Construction sequence of shield tunnel sections

Fig.5 Engineering design scheme

2. ***For the DEM part, calibration process of the soil parameters (for example, against the results from the triaxial tests) should be given.***

**Response：**

According to the expert opinion, the soil parameter test process of the paper is supplemented, and the specific content is as follows.

**In the DEM field, the contact parameters of particles are calibrated through a series of large-scale triaxial test analyses. In the process of numerical simulation in this paper, the selection of parameters is subject to field monitoring, and the calibration test is carried out by triaxial test, and the modeling parameters of the following soils are obtained. Triaxial test is a common method to determine the macroscopic parameters of soil. Unconsolidated and undrained test is carried out on a series of sand and clay with density gradient through the triaxial tester. The relationship between the internal friction Angle ϕ, cohesion force c, deformation modulus k and porosity is fitted, and the correlation between the shear strength and porosity of the soil is studied, which provides a basis for the parameter assignment of the subsequent shield model.**

**Triaxial analysis test**

3. ***In fact, DEM is not an appropriated numerical tool for modelling the process of tunnelling, given that it cannot mimicking simultaneous grouting. The soil loss rate is 6.15% based on the diameter of the tunnel excavation and the outer diameter of the segment. But adoption of this value will overestimate the disturbance tunnelling to the surrounding soil and pile foundation, as simultaneous grouting was not considered in the simulation.***

**Response：**

This paper will supplement the content of this part in the future research outlook. Synchronous grouting was not considered in the simulation. Although the selection of tunnel excavation diameter and pipe section outer diameter in this paper is subject to the actual situation, the simulation results will have certain deviations. In the future research, the numerical simulation of synchronous grouting process can be considered to improve the accuracy of simulation results.

4. ***The paper used FLAC2D and PFC2D to simulate the deformation process of pile foundation during tunnel driving. The tunnelling problem actually is an 3D problem; therefore, the limitation of the study should be highlighted in the discussions and conclusions.***

**Response：**

According to the expert opinion, the limitations of this study are supplemented in the conclusion of the article. This paper shows that although 2D method can be used to simulate surface deformation, there are still shortcomings in 3D simulation. Subsequently, FLAC3D and PFC3D can be used for numerical simulation to improve the accuracy of model simulation. In the last part of the article:

Firstly, the coupling models in this paper are all 2D methods. Although 2D methods can simulate deformation on a plane, in reality, the tunnelling process is a three-dimensional problem. In the future, researchers can use FLAC3D and PFC3D for coupling simulations.
