# Supplementary material for: Study on deformation of tunnel pile foundation based on discrete element method and finite difference method
Source: PLoS One. 2024 Jul 18;19(7):e0307405. doi: 10.1371/journal.pone.0307405 (PMC11257389; doi:10.1371/journal.pone.0307405)
Supplement: Attachment — Submitted filename: Response to Reviewers.docx [file pone.0307405.s003.docx]

**Dear Editors and Reviewers,**

**We sincerely thank our editors and all reviewers for their valuable feedback, which has helped us to improve the quality of our manuscripts. Reviewer comments are listed in bold font and numbered for reference, and our responses are given in normal font. Changes/additions to the original text are shown in red font.**

**Editor:**

**1) The abstract is lengthy. It must be shortened.**

We have condensed the abstract to 150 words. The revised abstract is as follows:

The deformation of pile caused by tunnel excavation will weaken the bearing capacity of the foundation. In order to investigate the deformation response of pile induced by the construction of three-hole small spacing tunnel, the DEM-FDM (discrete element method and finite difference method) coupling numerical simulation method were used to simulate the deformation process of pile during tunnel excavation. This paper probed into the deformation response of pile by three factors: the length of pile, the pile-tunnel spacing, and the three-hole tunnel construction. The results showed that as the pile-tunnel spacing decreases, the incremental horizontal displacement of the pile top became more significant when the three-hole tunnel was excavated. The excavation resulting in four zones of horizontal displacement concentration. The prominent locations were mainly concentrated on both sides of the tunnel and the ground directly above the tunnel. The research findings of this study can provide insights and references for the design and construction of shield tunneling underpassing piles.

**2) The introduction is still lengthy.**

We have further streamlined the language and removed redundant sentences from the manuscript.

**3) All features must be mentioned on the image of Figure 2. Moreover, it is too small.**

The main research approach employed in this study is numerical simulation analysis, focusing on the mechanism of repeated disturbances from closely spaced tunnels on adjacent piles. Figure 2 serves merely as a calibration experiment to validate the parameters used in the simulations, while the triaxial tests are conventional experimental methods. Therefore, the article does not extensively elaborate on their details. The original photograph for Figure 2 was taken earlier and the angle chosen was not ideal. We have opted to remove Figure 2, which does not compromise the integrity of the paper or subsequent analyses. We appreciate the editor's suggestions and efforts.

**4) All used material properties need references, especially for the data in Tables 1 and Tables 2. This comment was mentioned before and not addressed!**

We have supplemented the references according to the editor's suggestions to support the data presented in the tables. The data in Table 1 are derived from our own test results, with the cited references being previously published work by our team. The data in Table 2 are selected based on Chinese industry standards, and we have added the relevant standards as references.

**5) The verification of numerical results without any references or experiments has no meaning.**

This paper analyzes a typical working condition. Although three-line parallel small-spacing tunnels are rare, they do exist. Consequently, there are few prior studies on this topic, and the existing literature does not specifically address small-spacing tunnels. Therefore, this paper does not validate its results against existing literature. However, as subway construction expands, the frequency of such situations increases, making this study valuable for similar construction projects. In this paper, we primarily validate the model's reliability by comparing numerical simulation data with settlement data obtained from field monitoring (Figure 5). Similar studies in the existing literature also predominantly use monitoring data for model validation. The settlement curves obtained in this paper align well with the field-monitored settlement curves in terms of settlement area width and peak settlement, supporting further analysis in the paper. Additionally, for pile deformation patterns, the paper compares and explains the findings with those in references [27], [32], [30], [36] when analyzing Figures 8, 10, 11, 12, 15, and 19.

Fig.5 The results of numerical simulation verification

**6) Based on the first reviewing, the reviewer asked the authors to provide some engineering background for the three-hole small distance tunnel construction near pile foundation. It must be added to the main text.**

We have added the engineering background in the "Construction Plan Design" section of the paper. This includes basic information about the project, such as tunnel spacing, depth, diameter, soil type, and the actual construction sequence. Additionally, we have included a schematic diagram of the site tunnels. The revised content is as follows:

“Take an urban rail transit shield section in China as an example, the shield is a three-hole section, including the up line, the down line and the stop line. The designed start and end points for the uprunning line in this section are: SK20+226.052 to SK20+741.314, spanning a total length of 515.262 meters. Similarly, the designed start and end points for the downward line are: XK20+227.065 to XK20+741.330, covering a total length of 514.197 meters. The start and end points for the parking line in this section are: TK0+112.500 to TK0+627.514, with a total length of 515.014 meters. The vertical distance between the top and bottom lines is 22 meters. Therefore, this tunnel is a small clear distance tunnel. The maximum and minimum slope of the line are 4‰ and 2‰, respectively. The burial depth of the line ranges from 8.916 to 9.946 m, and the outer diameter of the segment is 6.2 m.

The line of the three-hole shield tunnel section travels along the existing road. Along the line are the city's main roads and important commercial blocks and residential areas. The underground pipeline along the line is dense and passes through the existing civil air defense tunnel. The actual scene diagram of the project case is shown in Fig. 4 (a).”

(a) Project case site layout

(b) Construction sequence of shield tunnel sections

Fig.4 Engineering design scheme

**7) No proper answer was mentioned for the comment of the reviewer that the DEM is not an appropriated numerical tool for modelling the process of tunnelling. References must be used and descriptions must be added to the main text.**

Numerical simulation of tunnel grouting has always been one of the challenging aspects of grouting technology research. However, some studies have successfully employed the Discrete Element Method (DEM) for the numerical analysis of filling grouting (see reference [32]). Additionally, DEM is very suitable for simulating discrete systems, such as sand and gravel soils, and can even simulate the cutting process during excavation. Therefore, it is widely used in tunnel construction-related research (see reference [30]; a search for "DEM" and "Tunnel" in Web of Science retrieves 382 related papers). The original intention of the reviewer was not to indicate that DEM is unsuitable for simulating tunnel excavation, but rather to emphasize that when using this method to analyze shield tunneling, it is important to appropriately address the issue of the tail gap. The introduction of the paper cites relevant literature to illustrate the prevalence of DEM in shield tunneling research, such as references [27], [30], [31], and [32]. Furthermore, the tunnels discussed in this paper are mainly excavated in sandy soils, and there is extensive research on using DEM to simulate the mechanical behavior of sandy soils. This paper specifically considers the advantages of DEM in simulating soil layers and the advantages of the finite element method(FEM) in simulating continuous media (concrete) to establish a numerical simulation model.

The reviewer pointed out that the article does not consider grouting, and therefore, using the theoretical ground loss rate might overestimate the impact of excavation on adjacent piles. The reviewer's comment is very insightful, and it is indeed an aspect that was not considered in this paper. Consequently, we have written a section in the conclusion that introduces considerations for further research based on this study, specifically mentioning the need to account for grouting when using DEM to study the impact of tunnel construction. On the other hand, since this paper primarily explores the influence of construction sequence, pile-tunnel distance, and pile length on the deformation of adjacent piles, the ground loss rate is kept constant for all conditions. Thus, the patterns discussed in the calculation results are not affected by the factors addressed in this paper. However, based on the reviewer's suggestion, we have added a supplement in the “DEM-FDM Coupling Model and Parameter Setting” section, explaining the basic assumptions and premises of our model. The specific content is as follows:

**Introduction:**

“Discrete element method (DEM), as an emerging numerical simulation method, have been applied in various fields[28–31]. In geotechnical engineering, it can simulate the interaction between particles or particle groups, and is suitable for simulating the behavior of granular materials such as soil. FDM, as an continuous medium method, can better describe the changes in continuous media and is more suitable for concrete continuity modeling. By coupling this two methods, the interaction between granularity and continuity can be considered comprehensively, so that the behavior of pile in the process of excavation can be simulated more accurately. The DEM-FDM coupling method was verified by Gholaminejad et al [26]. It is a robust method to simulate geotechnical problems. lee et al. [27] used DEM and FDM to conduct 3D numerical simulation of earth pressure balance (EPB) shield tunnel. The simulation results show that the numerical model based on DEM-FDM coupling can reasonably simulate tunnel shield tunneling under the condition of TBM operation, and has good robustness. Consequently, more and more research has based on DEM-FDM coupling method[28-30]. Qu et al. [31] established a computational model using the coupled DEM-FDM method and realized the simulation of the whole process of shield construction. By using the coupling method, better results can be obtained in the study of pile and tunnel[32,33].”

**DEM-FDM coupling model and parameter setting:**

“In order to investigate the soil deformation caused by subway tunnel excavation and the response of pile foundation deformation, this paper selects the finite difference program FLAC2D to build tunnel segments and adjacent pile foundation through grid modeling. PFC2D is used to deposit strata through particles. As the problems in computational efficiency of 3D computational analysis have not been solved, this paper currently discusses the horizontal deformation of piles only at the 2D level. A two-dimensional computational stratigraphic model with a width of 72m and a height of 26m was established, as shown in Fig. 1. The diameter of the tunnel excavation is 6.4m, the outer diameter of the segment is 6.2m, that is, the shield tail clearance is 10cm, the soil loss rate is 6.15%, and the thickness of the segment is 35cm according to the actual construction situation. In the process of accumulation modeling, in order to ensure the accuracy of particle part calculation. The number of particles should be reduced as much as possible to ensure that the computer can give full play to the calculation power. The particle size is 5cm, and the total number of particles before excavation is 84,117. In order to reflect the influence mechanism of buried depth of three-wire tunnel with small distance on pile foundation and stratum, the tunnel buried depth is set as 13.1m, the covering layer thickness at the top of the tunnel is 10m, the distance between the bottom of the segment and the bottom of the model is 10m (1.6D), and the distance between the outer side of the tunnel segments on both sides of the model is 25m (4D). It can ensure that the effect of model boundary does not affect the calculation result [34]. This chapter only discusses the geometric characteristics of pile foundation and the influence of the distance between pile foundation and tunnel, so the difference of different soil layers is ignored. The horizontal displacements of the piles obtained from the calculations may be large compared to the actual conditions because the effect of infill grouting on the soil loss rate was not considered. However, this does not affect the analysis of the effect of pile length and pile-tunnel spacing since the fill grouting was not considered for all the conditions.”

**Discussions:**

“This paper puts forward some meaningful conclusions, however, there are still some limitations. Firstly, the coupling models in this paper are all 2D methods. Although 2D methods can simulate deformation on a plane, in reality, the tunnelling process is a three-dimensional problem. In the future, researchers can use FLAC3D and PFC3D for coupling simulations. Secondly, synchronous grouting is not considered in the simulation process of this paper. Although the selection of tunnel excavation diameter and segment outside diameter in this paper is subject to the actual situation, there will be some deviation in the simulation results. In the future research, the numerical simulation of synchronous grouting process can be considered to improve the accuracy of simulation results. Finally, in the future, researchers can supplement the effects of soil properties, groundwater conditions, and tunnel construction methods to improve the depth and breadth of research.”

**8) Again, the reviewer asked to mention the limitation of the study should be highlighted in the discussions and conclusions. Not done properly.**

Thank you to the reviewers and the editor for your suggestions. In the previous round of review, the reviewers highlighted the article's limitations, specifically the lack of consideration for grouting and the influence of thickness direction, suggesting the need for further 3D computational analysis. We have already addressed the grouting issue in our previous response and included relevant discussions in the final paragraph of the conclusion. Regarding the 3D analysis, conducting large-scale DEM analyses in three dimensions requires substantial computational power and long computation times. Therefore, most engineering analyses using DEM in the literature primarily focus on 2D calculations. Although 3D computation is the ultimate goal in this field and we are actively exploring ways to enhance computational efficiency, it will only be reflected in our future research outcomes. Following the reviewers' suggestions, we have discussed these limitations in the final paragraph of the conclusion. Additionally, we have included relevant explanations in the "DEM-FDM Coupling Model and Parameter Setting" section to address readers' considerations when reviewing this paper. The specific content is as follows:

**DEM-FDM coupling model and parameter setting:**

“In order to investigate the soil deformation caused by subway tunnel excavation and the response of pile foundation deformation, this paper selects the finite difference program FLAC2D to build tunnel segments and adjacent pile foundation through grid modeling. PFC2D is used to deposit strata through particles. As the problems in computational efficiency of 3D computational analysis have not been solved, this paper currently discusses the horizontal deformation of piles only at the 2D level. A two-dimensional computational stratigraphic model with a width of 72m and a height of 26m was established, as shown in Fig. 1. The diameter of the tunnel excavation is 6.4m, the outer diameter of the segment is 6.2m, that is, the shield tail clearance is 10cm, the soil loss rate is 6.15%, and the thickness of the segment is 35cm according to the actual construction situation. In the process of accumulation modeling, in order to ensure the accuracy of particle part calculation. The number of particles should be reduced as much as possible to ensure that the computer can give full play to the calculation power. The particle size is 5cm, and the total number of particles before excavation is 84,117. In order to reflect the influence mechanism of buried depth of three-wire tunnel with small distance on pile foundation and stratum, the tunnel buried depth is set as 13.1m, the covering layer thickness at the top of the tunnel is 10m, the distance between the bottom of the segment and the bottom of the model is 10m (1.6D), and the distance between the outer side of the tunnel segments on both sides of the model is 25m (4D). It can ensure that the effect of model boundary does not affect the calculation result [34]. This chapter only discusses the geometric characteristics of pile foundation and the influence of the distance between pile foundation and tunnel, so the difference of different soil layers is ignored. The horizontal displacements of the piles obtained from the calculations may be large compared to the actual conditions because the effect of infill grouting on the soil loss rate was not considered. However, this does not affect the analysis of the effect of pile length and pile-tunnel spacing since the fill grouting was not considered for all the conditions.”

**Discussions:**

“This paper puts forward some meaningful conclusions, however, there are still some limitations. Firstly, the coupling models in this paper are all 2D methods. Although 2D methods can simulate deformation on a plane, in reality, the tunnelling process is a three-dimensional problem. In the future, researchers can use FLAC3D and PFC3D for coupling simulations. Secondly, synchronous grouting is not considered in the simulation process of this paper. Although the selection of tunnel excavation diameter and segment outside diameter in this paper is subject to the actual situation, there will be some deviation in the simulation results. In the future research, the numerical simulation of synchronous grouting process can be considered to improve the accuracy of simulation results. Finally, in the future, researchers can supplement the effects of soil properties, groundwater conditions, and tunnel construction methods to improve the depth and breadth of research.”

**Reviewer #3:**

**1. How does the DEM-FDM coupled numerical simulation method, utilizing software such as FLAC2D and PFC2D, contribute to understanding the deformation response mechanism of pile foundations during tunnel construction, specifically in the context of a three-hole small clear distance tunnel?**

The Finite Difference Method (FDM) and Finite Element Method (FEM), due to their high computational efficiency, are widely used for simulating continuous materials like concrete and steel. These methods yield good computational results for such materials. The Discrete Element Method (DEM), on the other hand, assumes materials as assemblies of particles and can create particles of different gradations and shapes, making it highly suitable for simulating soil or large rock masses. Therefore, this paper employs a DEM-FDM coupled approach for modeling and analysis, leveraging the advantages of both methods to achieve excellent computational results. Additionally, this coupled computation method is relatively new and is gaining increasing attention from scholars, thus offering innovation in research methodology. This approach is also more accurate than using FDM alone, as it accounts for the separation between soil particles and the pile, as well as the process from separation to filling between the soil and the segments. This allows for a more detailed reproduction of the repeated impact of three-hole tunnel excavation on the soil.

**2. What were the key findings regarding the influence of different factors, such as the length of the pile foundation, the distance between the tunnel and pile foundation, and the small clear distance of the three-hole tunnel construction, on the deformation response of pile foundations? How did these factors affect vertical deformation, settlement displacement, and uplift peak values?**

Thank you to the reviewers for their suggestions. The impact of the three influencing factors on the displacement and stress of the pile is as follows. The original conclusions and corresponding analysis logic were indeed not clear enough, and we have now made the necessary revisions.

In our analysis of the impact of pile length, we found that tunnel construction significantly affects the horizontal displacement of piles, primarily concentrated at the tunnel's burial depth. Therefore, when the pile length is short, the horizontal displacement peak is located at the pile bottom, while for longer piles, the peak is located in the middle of the pile. Piles with a length of H+D are more sensitive to this impact, mainly exhibiting inclined deformation. However, when the pile length is H+2D, the pile foundation tends to bend, resulting in differences in axial stress between the two. Particularly when the pile-tunnel distance is small, the axial stress is lower for short piles and higher for long piles. When the pile-tunnel distance is 1D and the pile length is H+D, the pile top moves 5.3mm towards the tunnel, while the pile bottom moves 4.9mm away from the tunnel.

Our analysis of the pile-tunnel distance indicates that the influence range of tunnel construction on the horizontal displacement of piles is approximately 2.5D, with the horizontal displacement of the pile top showing a linear correlation with the pile-tunnel distance. Within a 3D range, the vertical displacement of the pile also exhibits a good linear correlation with the pile-tunnel distance. Additionally, for long piles, a decrease in pile-tunnel distance leads to bending of the pile, resulting in additional axial stress. When the pile-tunnel distance is 1D and the pile length is H+2D, the peak axial stress in the tunnel reaches 1MPa.

After analyzing the effects of construction in narrow-spaced tunnels, we found that for the pile top, the horizontal displacement decreases gradually with the completion of each of the three tunnels, with the maximum displacement occurring after the first tunnel's construction. However, for the horizontal displacement at the pile bottom, it increases gradually as each of the three tunnels is completed. Analyzing Figure 19(c), we observed that long piles exhibit an initial increase followed by a decrease in horizontal displacement at the pile bottom as each tunnel is completed in close spacing conditions. This phenomenon becomes less pronounced with increasing spacing. In terms of axial stress on the piles, long piles in close spacing are more sensitive to repeated disturbances from tunnel construction.

The modified content is as follows:

**“Effect of pile length”**

Fig. 8 shows the horizontal displacement of pile top and pile bottom of three different lengths of piles at different distances between tunnel and pile. In the figure, the positive value of horizontal displacement is the displacement away from the tunnel direction, and the negative value of horizontal displacement means that the pile foundation moves towards the tunnel. Fig. 8 shows that the horizontal displacement of adjacent pile foundation induced by the construction of three-line small clear distance tunnel is much larger than the vertical displacement. When the distance between three kinds of pile foundation and tunnel is 3D, the horizontal displacement values of pile top and pile bottom are relatively close. However, when the distance between pile foundation and tunnel is 1D and 1.5D, the horizontal displacement difference between pile top and pile bottom caused by tunnel construction is large. When the distance between the tunnel and the pile is 1D, for the pile with the pile length H+D, the pile top moves 5.3mm towards the tunnel and the pile bottom moves 4.9mm away from the tunnel. The piles with length H+D are more sensitive to this effect, at this time the pile is mainly inclined to deformation, but when the pile length is H+2D there is a tendency of bending of the pile. For the cases set in this chapter, the pile foundation with pile length H+D is the most dangerous when the distance between the pile foundation and the tunnel is less than 1.5D [36].

As can be seen in Fig. 11 (a), when the distance between pile foundation and tunnel is 1D, long piles (pile length H+2D and H+D) mainly show bending. At this time, the axial stress near the tunnel side of the pile side reaches 1MPa, which is manifested as compressive stress, while the axial stress away from the tunnel side reaches 600kPa, which is manifested as tensile stress. Short piles (pile length: H) are mainly characterized by inclination of the pile base and therefore axial stresses are low. A comparative analysis of Fig. 11 (a) to Fig. 11 (c) also shows that as the distance between pile foundation and tunnel increases, the axial stress difference on both sides of pile foundation also gradually decreases, which also indicates that the influence of tunnel construction on pile foundation is gradually decreasing [30].

**“Effect of pile-tunnel spacing”**

In this section, the deformation response of adjacent pile foundation caused by tunnel construction is calculated and analyzed under 5 kinds of spacing between pile foundation and tunnel. The influence of tunnel construction on adjacent pile foundation is mainly manifested as the horizontal deformation of pile foundation. By comparison and analysis of the five kinds of spacing in Fig. 12, it can be found that the horizontal displacement of pile top begins to be less than 1mm after the distance between pile and tunnel is greater than 2.5D. At this time, the horizontal displacement of the pile body at the same position as the buried depth of the tunnel is only 1.5mm. It shows that when the distance between tunnel and pile is greater than 2.5D, the influence of tunnel construction on pile foundation is very weak [27].

Fig. 13 shows the corresponding relationship between the horizontal displacement of pile top and pile bottom of three lengths and the distance between tunnel and pile foundation. It can be seen that as a whole, with the increase of the distance between pile foundation and tunnel, the horizontal displacement of pile top and pile bottom gradually decreases. For the top of piles, the horizontal displacement of the top of piles of three lengths is not significant. There are some differences in the sensitivity of distance between tunnel and pile foundation. In contrast, the horizontal displacement of pile bottom shows a good linear correlation with the distance between tunnel and pile, in which the horizontal displacement of pile with pile length H+2D is not affected by the distance between tunnel and pile, because the pile bottom of such long piles is no longer in the concentration area 4 of horizontal displacement caused by tunnel construction.

The deep-buried soil mass within the tunnel experiences compression from the overlying soil mass and pipe segments, leading to a migration away from the tunnel's direction. This migration results in the bending of adjacent pile foundations. In a model extending H+2D in length, it becomes apparent that when the distance between the tunnel and the pile is 1D, the pile foundation showcases a displacement of 4.5mm away from the tunnel at the tunnel's depth. Simultaneously, the pile top displays a displacement of 5mm toward the tunnel. However, the pile's end doesn't exhibit significant horizontal displacement, yet all three segments distinctly exhibit bending.

**“Effect of Three-Line Tunnel Construction on Pile”**

Fig. 18 shows the horizontal displacement of pile top of three lengths of pile foundation at different excavation stages, and the horizontal coordinate is the three stages completed by three tunnels in turn. The horizontal displacement of pile top increases gradually with the three lines penetrating one by one. The farther the tunnel is from the pile foundation, the less significant the increment of pile top horizontal displacement caused by the three-hole tunnel. For pile foundation with pile length L2 (H+D), when the distance between pile and tunnel is 3D, the horizontal displacement increment of pile top is less than 1.6mm with the penetration of three tunnels one by one. For pile foundation with pile length L3(H+2D), when the distance between pile and tunnel is 2.5D and 3D, the incremental horizontal displacement of pile top caused by double hole penetration is less than 0.5mm. However, when the three holes are connected, the incremental horizontal displacement of pile top is relatively larger, about 1-1.5mm, which is also reflected in Fig. 18 (b) and Fig. 18 (a).

Fig. 19 shows the horizontal displacement of pile bottom for three lengths of pile foundation at different excavation stages. The horizontal positions of the pile bottoms of the three kinds of piles do not show significant positive correlation with the three kinds of tunnels penetrating one by one. The reason is that when only two lines are connected, the pile foundation is relatively in the horizontal displacement concentration area on both sides of the tunnel. However, when the third line is through, if the distance between pile foundation and tunnel is very close, the position of pile bottom is below the horizontal displacement concentration area on both sides of the tunnel. Therefore, the horizontal displacement does not increase, and even decreases [34]. For long pile at small spacing, the horizontal displacement at the pile bottom first increases and then decreases as the three tunnels are sequentially completed. This phenomenon becomes less noticeable as the spacing increases.

**“Conclusions”**

The impact of tunnel construction on the horizontal displacement of pile is significant, primarily concentrated at the depth of the tunnel. During tunnel construction, short pile tend to tilt, while long pile exhibit a bending trend. Pile with a length of H+D are most sensitive to tunnel construction. The axial stress of short pile shows little change when they tilt, whereas the axial stress of long pile increases when they bend.

The influence range of tunnel construction on pile is approximately 2.5D. Within a range of 3D, the horizontal displacement of the pile top and bottom shows a linear correlation with the pile-tunnel distance. A decrease in the pile-tunnel distance causes long pile to bend. When the pile-tunnel distance is 1D and the pile length is H+2D, the peak axial stress of the tunnel reaches 1MPa.

At small pile-tunnel distances, the horizontal displacement of the pile top gradually decreases as the three tunnels are sequentially constructed, with the maximum displacement occurring after the completion of the first tunnel. The horizontal displacement at the bottom of short piles gradually increases, while that of long pile first increases and then decreases. The axial stress of long pile is more sensitive to the repeated disturbances of the three-tunnel construction.

**3. Can you elaborate on the observations made regarding the impact of varying pile lengths on the vertical deformation caused by tunnel construction and how settlement displacement and uplift peak values were distributed along the pile foundation in different scenarios?**

Thank you for the suggestions from the reviewer. We have revised the section to improve its logical coherence and focus. The revised content is as follows:

“Fig. 6 shows the vertical displacement of the pile top and bottom under different conditions. The results indicate that tunnel construction can cause settlement or slight uplift of the piles. When the pile-tunnel distance is 1D, the pile with a length of H+D experiences the maximum settlement, which is only 3mm. Thus, the vertical displacement of the pile caused by tunnel construction is not significant. Fig. 6 demonstrates that when the pile-tunnel distance is small, there is a slight difference in vertical displacement between the top and bottom of long piles, whereas the vertical displacement of the top and bottom of short piles is almost equal. This indicates that long piles undergo slight compressive deformation, while short piles experience overall settlement.”

“Fig. 7 shows the contour of vertical displacement for pile and the surrounding soil when the pile-tunnel distance is 1D, for three different pile lengths. The results indicate that the vertical displacement at the pile top is greater than at the pile bottom. For short piles (when the pile length is less than H+D), the vertical displacement of the pile gradually decreases from the pile top to the pile bottom. However, for long piles, the vertical displacement is smallest at the tunnel depth and gradually increases below the tunnel depth.”

|  |  |
| --- | --- |
| Fig. 6 Vertical displacement of pile foundation with different distance between three kinds of pile foundation and tunnel. | Fig. 7 Vertical displacement cloud mapof three pile foundation models (m) |

**4. How did the distance between the tunnel and pile foundation influence horizontal displacement differences between the pile top and bottom, and what implications did this have for the structural behavior and integrity of the pile foundation under tunnel construction loads?**

Thank you for the reviewer's comments. Firstly, revisions to Figure 13 in the manuscript indicate that there are differences in horizontal displacements between the pile top and bottom, which decrease as the distance between the pile and tunnel increases. Further comparison with Figure 14 reveals that short piles primarily exhibit tilting deformation, while long piles exhibit bending deformation. Moreover, as the pile-tunnel distance decreases, the degree of tilting increases for short piles, and the bending extent increases for long piles. In service, tilting of short piles is detrimental to the pile top, whereas bending of long piles induces additional axial stresses in the pile body. Figure 11 demonstrates that there are different additional stresses on the sides near and away from the pile, further confirming the bending tendency of the piles. Such stresses may cause cracking of the concrete protection layer on the steel bars, thereby increasing the risk of steel corrosion in groundwater. Overall, these findings are unfavorable for pile performance. We have adjusted the manuscript accordingly and supplemented additional content.

**“Effect of pile length”**

As can be seen in Fig. 11 (a), when the distance between pile foundation and tunnel is 1D, long piles (pile length H+2D and H+D) mainly show bending. At this time, the axial stress near the tunnel side of the pile side reaches 1MPa, which is manifested as compressive stress, while the axial stress away from the tunnel side reaches 600kPa, which is manifested as tensile stress. This tensile stress could potentially cause cracking of the concrete protective layer on the surface of the steel bars, thereby increasing the risk of underground water corroding the reinforcement bars. Short piles (pile length H) are mainly characterized by inclination of the pile base and therefore axial stresses are low. a comparative analysis of Fig. 11 (a) to Fig. 11 (c) also shows that as the distance between pile foundation and tunnel increases, the axial stress difference on both sides of pile foundation also gradually decreases, which also indicates that the influence of tunnel construction on pile foundation is gradually decreasing [30].

**“Effect of pile-tunnel spacing”**

“Fig. 13 shows the corresponding relationship between the horizontal displacement of pile top and pile bottom of three lengths and the distance between tunnel and pile foundation. It can be seen that as a whole, with the increase of the distance between pile foundation and tunnel, the horizontal displacement of pile top and pile bottom gradually decreases. There exists a difference in horizontal displacement between the pile top and bottom, which decreases as the distance between the pile and tunnel increases. There are some differences in the sensitivity of distance between tunnel and pile foundation. In contrast, the horizontal displacement of pile bottom shows a good linear correlation with the distance between tunnel and pile, in which the horizontal displacement of pile with pile length H+2D is not affected by the distance between tunnel and pile, because the pile bottom of such long piles is no longer in the concentration area 4 of horizontal displacement caused by tunnel construction.”

“The deep-buried soil mass within the tunnel experiences compression from the overlying soil mass and pipe segments, leading to a migration away from the tunnel's direction. This migration results in the bending of adjacent pile foundations. In a model extending H+2D in length, it becomes apparent that when the distance between the tunnel and the pile is 1D, the pile foundation showcases a displacement of 4.5mm away from the tunnel at the tunnel's depth. Simultaneously, the pile top displays a displacement of 5mm toward the tunnel. However, the pile's end doesn't exhibit significant horizontal displacement, yet all three segments distinctly exhibit bending. As the pile-tunnel spacing decreases, the tilting extent of short pile gradually increases, while the bending extent of long pile also gradually increases. For pile in service, the tilting of short pile is detrimental to the pile cap, whereas the bending of long pile induces additional axial stresses in the pil shaft.”

|  |  |
| --- | --- |
| Fig. 11 Axial stress distribution of three piles at different distances: (a)1D | Fig. 13 Horizontal displacements of three kinds of piles with different spacing |
|  | |
| Fig. 14 Distribution of horizontal displacements along the axis of piles at different distances: (c) The pile length is L3 | |

**5. What were the critical indications from the study regarding the distribution of axial stress on the pile body for short pile foundations and how did the incremental horizontal displacement at the pile head vary as the pile foundation approached the tunnel during the construction of the three-hole closely spaced tunnels?**

Thank you for the reviewer's question. Through Figures 11 and 16, it can be observed that due to tilting predominantly occurring in short piles and bending in long piles, the axial stresses generated in short piles are not significant, whereas those in long piles merit greater attention. Figure 11 indicates that for a pile of length H+2D and a small pile-tunnel distance, axial stresses include compressive stress of 1 MPa on the side near the tunnel and tensile stress of 500 kPa on the side away from the tunnel, with the maximum axial stress occurring at the tunnel depth. We emphasize that the critical aspect of axial stress in long piles is the magnitude of tensile stress, as this can lead to cracking of the concrete protection layer on the pile's steel reinforcement, thereby increasing the risk of steel corrosion in groundwater.

Thank you for your suggestion. Figure 13 indicates that as the distance between pile and tunnel increases, the horizontal displacement of the pile top gradually decreases. However, compared to the linear relationship observed at the pile bottom, the variation in horizontal displacement at the pile top does not strictly follow a linear pattern. Specifically, the non-linearity of horizontal displacement at the pile top is more pronounced for short piles, whereas long piles exhibit a higher degree of linearity in horizontal displacement at the pile top.

|  |  |
| --- | --- |
| Fig. 11 Axial stress distribution of three piles at different distances: (a)1D | Fig. 13 Horizontal displacements of three kinds of piles with different spacing |

**6. How did the construction of three-hole closely spaced tunnels impact soil deformation in the horizontal direction, leading to the formation of distinct zones of horizontal displacement concentration? Which areas around the tunnel were identified as prominent locations for horizontal displacement concentration based on the study findings?**

Thank you for your inquiry. According to Figures 9 and 17, tunnel construction induces horizontal deformations in the surrounding soil. The horizontal deformation characteristics of the soil mainly manifest as convergence of the soil on the left and right sides of the tunnel, accompanied by corresponding horizontal deformations at the ground surface. For the three-line tunnel configuration, we observed that as each of the three tunnel sections is excavated sequentially, the horizontal displacement of the ground surface gradually increases. We identified four concentrated areas of horizontal displacement along the cross-section, located respectively on the outer sides of the two tunnel sections and directly above them. When the pile-tunnel spacing is small, the pile top is often affected by Area 2, while the pile at the tunnel depth is influenced by Area 4.

|  |  |
| --- | --- |
| Fig. 9 Horizontal displacement cloud image at different pile lengths | Fig. 17 Cloud map of horizontal displacement of adjacent pile foundation and stratum induced by tunnel construction |

**7. In what ways can the research outcomes presented in this study be applied to inform and improve the design and construction practices for shield tunneling projects that involve underpassing pile foundations? What considerations should engineers and practitioners take into account to mitigate deformation risks and ensure the safety and stability of pile-supported structures during tunnel construction activities?**

Thank you for your comments. This study investigates the effects of construction of a three-line close-spacing tunnel on nearby piles in terms of displacement and stress. We analyzed the influence of pile-tunnel spacing, pile length, and the construction process of the three-line tunnel. The original manuscript indeed lacked specific recommendations on effective construction measures. Following the reviewer's suggestions, we have included design considerations and construction recommendations in the section titled "Mitigation measures and design considerations". We hope these suggestions will assist practitioners in minimizing adverse impacts of tunnel construction on nearby piles.

**“Mitigation measures and design considerations”**

The vibration and deformation of pile foundation caused by tunnel construction may affect the structural stability and soil mechanical properties. These impacts may require a range of measures to manage and mitigate before, during and after construction. The foregoing study discussed the pile foundation response of the three-cave tunnel under different conditions. This section will take relevant mitigation measures to cause stratum disturbance and soil deformation of the three-cave tunnel, so as to reduce the impact on the pile foundation, and put forward mitigation suggestions.

1. Geological investigation and analysis: carry out detailed geological investigation before construction to understand the formation conditions and geological characteristics. This helps to predict possible geological changes and the impact of tunnel construction on the soil layer.

2. Structural design optimization: By optimizing the structural design of the tunnel, the impact on the surrounding soil and pile foundation can be reduced. This may include adjusting the depth, width and shape of the tunnel to reduce formation disturbance.

3. Use appropriate construction techniques: Grouting is a critical process in shield tunneling that efficiently fills gaps behind the shield. The deformation of pile induced by tunnel excavation is primarily horizontal at the depth of the tunnel. Therefore, adjusting the grouting pressure and volume on the side of the tunnel facing the piles can reduce additional displacement of the pile.

4. Formation reinforcement and support measures: Take formation reinforcement and support measures around the tunnel, such as the use of supporting piles, soil reinforcement or other geological engineering techniques to protect the pile foundation from disturbance.

5. Monitoring and adjustment: real-time monitoring during the construction process, and adjustment and control according to the monitoring results. This helps to identify problems in a timely manner and take necessary corrective actions to reduce negative impacts.

**Reviewer #4:**

The paper presents a study to report on the deformation patterns of nearby piles during construction of a three-holes, small-spaced subway tunnel. The paper is well written and the results are presented with proper justifications. The problem is often encountered in practice and therefore has the potential for publication on the condition that some clarifications are provided in the revised manuscript.

**1. Spaces should be added between figures and units in the manuscript.**

Based on expert advice, check the full text and add Spaces between figures and units.

**2. The manuscript uses “1.6D” as well as “4D” in lines 108 and 109, and an explanation of what parameter D stands for should have been provided at the time of its first appearance.**

In this article, the "D" represents the tunnel diameter, which in this case is 6.2m. According to expert opinion, when "6.2D" appears for the first time in this article, the explanation of "D" was added.

**3. In Figure 1 in the manuscript, the dimensional labeling on the upper right side of the model does not provide specific values.**

According to the expert opinion, add the size label in the upper right corner of Figure 1, and the revised figure 1 was as follows:

**Fig. 1 DEM - FDM coupling calculation model of shield tunnel**

**4. Velocity should be written as “m/s” or “m·s-1”. Some units in the manuscript are written in error, e.g., line 127, where “m·s-1” should not be used, and the authors are advised to verify this.**

According to expert opinions, check the full text, and modify the speed unit was m/s.

**5.In lines 213 and 214 of the manuscript, “9.946 meters” and “6.2 meters” should be written as “9.946 m” and “6.2 m”.**

According to expert opinions, simplify the expression of the article, modify the writing as "9.946m" and "6.2m".

**6.The first letter of the vertical label in figure 6 should be capitalized, and “surface settlement” should be changed to “Surface settlement”.**

According to the expert opinion, author drew the picture, and the revised picture is as follows:

Fig. 5 The results of numerical simulation verification

**7. Lines 372 and 373 are too long as section headings for the article, and it is suggested that they be revised to read: “The influence of tunnel-pile spacing on the response of pile”. Lines 455 and 456 are too long as section headings for the article, and it is suggested that they be revised to read: “The effect of repeated excavation on pile”.**

According to the opinions of experts, the sub-titles of the article are simplified and modified as follows: "Effect of Pile-tunnel Spacing" and "Effect of Three-Line Tunnel Construction on Pile".
